# Salbutamol attenuates arrhythmogenic effect of aminophylline in a hPSC-derived cardiac model

**DOI:** 10.1038/s41598-024-76846-4

**Published:** 2024-11-09

**Authors:** Daniil Kabanov, Simon Vrana Klimovic, Deborah Beckerová, Martin Molcan, Martin Scurek, Kristian Brat, Marketa Bebarova, Vladimir Rotrekl, Jan Pribyl, Martin Pesl

**Affiliations:** 1 CEITEC MU, Masaryk University, Brno, Czech Republic; 2 Department of Biology, Faculty of Medicine, Masaryk University, Brno, Czech Republic; 3 Department of Biochemistry, Faculty of Science, Masaryk University, Brno, Czech Republic; 4 Department of Respiratory Diseases, University Hospital Brno and Faculty of Medicine, Masaryk University, Brno, Czech Republic; 5 International Clinical Research Center, St. Anne’s University Hospital, Brno, Czech Republic; 6 First Department of Internal Medicine – Cardioangiology, Faculty of Medicine, Masaryk University, St. Anne’s University Hospital, Brno, Czech Republic; 7 Department of Physiology, Faculty of Medicine, Masaryk University, Brno, Czech Republic

**Keywords:** Salbutamol, Aminophylline, Atomic force microscopy, iPSC, HESC, Cardiomyocytes, Pulmonary drug screening, Drug cardiotoxicity, Biomechanical properties, Arrhythmogenic effects, Cell biology, Molecular biology, Cardiology, Nanoscience and technology

## Abstract

The combination of aminophylline and salbutamol is frequently used in clinical practice in the treatment of obstructive lung diseases. While the side effects (including arrhythmias) of the individual bronchodilator drugs were well described previously, the side effects of combined treatment are almost unknown. We aimed to study the arrhythmogenic potential of combined aminophylline and salbutamol treatment in vitro. For this purpose, we used the established atomic force microscopy (AFM) model coupled with cardiac organoids derived from human pluripotent stem cells (hPSC-CMs). We focused on the chronotropic, inotropic, and arrhythmogenic effects of salbutamol alone and aminophylline and salbutamol combined treatment. We used a method based on heart rate/beat rate variability (HRV/BRV) analysis to detect arrhythmic events in the hPSC-CM based AFM recordings. Salbutamol and aminophylline had a synergistic chronotropic and inotropic effect compared to the effects of monotherapy. Our main finding was that salbutamol reduced the arrhythmogenic effect of aminophylline, most likely mediated by endothelial nitric oxide synthase activated by beta-2 adrenergic receptors. These findings were replicated and confirmed using hPSC-CM derived from two cell lines (CCTL4 and CCTL12). Data suggest that salbutamol as an add-on therapy may not only deliver a bronchodilator effect but also increase the cardiovascular safety of aminophylline, as salbutamol reduces its arrhythmogenic potential.

**Supplementary Information:**

The online version contains supplementary material available at 10.1038/s41598-024-76846-4.

## Introduction

Bronchodilator drugs were designed to relieve symptoms of obstructive respiratory diseases (e.g., asthma and chronic obstructive pulmonary disease, COPD) by relaxing the smooth muscle apparatus of the lower airways. To date, three main classes of bronchodilators have been introduced in clinical practice, namely beta-2 agonists, anticholinergics, and methylxanthines^1^. These drugs are often used in combination, either in the stable phase of the disease or during asthma or COPD exacerbation^1–3^.

Cardiac adverse events are among the most frequent side effects of beta-2 agonists and methylxanthines. While the pro-arrhythmic activity of aminophylline is well described in the clinical setting^4,5^ and has recently been examined in our experimental study^6^, there is limited evidence on the effect and adverse events of these two bronchodilator classes used in combination^7–9^.

Salbutamol (also known as albuterol) is the most frequently used short-acting beta-2 agonist (SABA). A wide variety of side effects have been described with salbutamol treatment, especially when the drug is injected into the bloodstream^10^. Considering its effects on the heart, positive bathmotropic, chronotropic, and inotropic effects were described^11–13^. Salbutamol-induced hypokalemia may also affect cardiac function^14^ Serious arrhythmias (even ventricular fibrillation) in patients with preexisting heart diseases were reported^14,15^.

Using our established and previously described in vitro three-dimensional model of cardiac tissue formed by clusters of human pluripotent stem cells-derived cardiomyocytes (hPSC), we aimed to evaluate the effect of salbutamol alone and in combination with aminophylline on cardiac excitability and inotropy with a focus on their impact on arrhythmogenesis^6,16–20^.

## Materials and methods

### Cell cultivation

hESC line “center for cell therapy line” CCTL14 (46 XX) and CCTL12 (46 XX) derived at Masaryk University, Brno, and previously characterized^21^ was routinely maintained on a feeder layer of mitotically inactivated mouse embryonic fibroblasts. For formation of embryonic bodies (EB), hESC colonies were collected 4 days after seeding and subsequently broken down into smaller clumps which were seeded into EB medium (86% KO DMEM, 10% FBS, 1% L-glutamine, 1% penicillin/streptomycin, 1% nonessential amino acids, 0,1mM 2-mercaptoethanol and 10 µg/ml ascorbic acid) with 10 ng/ml BMP4 (R&D) and placed in hypoxic conditions (5% O_2_, 5% CO_2_) where they spontaneously formed EBs. After three days, the medium was removed and replaced with fresh EB medium supplemented with 5 ng/ml FGF2 (Peprotech), 10 ng/ml BMP4, and 6 ng/ml Activin A (R&D) for a four-day incubation. A three-day incubation followed this in EB media supplemented with 10 ng/ml VEGF (R&D) and 10 µM IWR1 (R&D). The following induction media was EB medium supplemented with 10 ng/ml VEGF and 5 ng/ml FGF2 with a media exchange every four days. After four days in this media (day 14 of differentiation), the EBs were transferred into a normoxic incubator (21% O_2_, 5% CO_2_) for the remaining 8 days of this induction period. From then onward, EBs remained in normoxia and were fed with EB medium every four days. 1 month old EBs with spontaneous contraction activity were used for analysis. Beating EBs were selected and transferred on a gelatin-coated PM3 dish to adhere to for measurement. These cellular constructs of cardiac syncytium were coupled to an AFM force sensor to perform a high-fidelity contraction pattern as an hPSC-CM-based biosensor^22^.

### Atomic force microscopy measurements

Contraction of beating EBs was assessed by Nanowizard 3 (Bruker-JPK) AFM combined with inverted light microscope IX-81 (Olympus) as previously described^6,17–19^. Liquid media was preheated to 37 Celsius degree and let to stabilize for at least 10 min before each measurement. Temperature was maintained unchanged during all following measurements^18,22^. A soft silicon nitride cantilever (Nitra-tall B, AppNano) was landed on EB attached to the petri dish (TPP). Real-time changes of force in time applied by contracting EB were recorded while administrating salbutamol (Fagron), its mixes with aminophylline (Fagron) in different concentrations, or butoxamine (Sigma Aldrich). Each measurement was preceded by initial equilibrium in Tyrode solution (composition in mM: NaCl 135, KCl 5.4, MgCl_2_ 0.9, CaCl_2_ 1.8, HEPES 10, NaH_2_PO_4_ 0.33, and glucose 5.5; pH 7.4 adjusted with 3 M NaOH), after which increasing concentrations of salbutamol (10 nM, 100 nM, 1 µM, 10 µM), mixes of salbutamol (S) and aminophylline (A) (10–1000 nM salbutamol, 10–100 µM aminophylline) or butoxamine (3 µM) was added^16^. All stock solutions of substances and treatments were prepared using Tyrode buffer, as both compounds show excellent water solubility (approx. 0.2 M for both), and no stock solutions in organic solvents were needed. Mixes were defined based on previous results of individual measurements of salbutamol in this article and aminophylline in our group’s previous article^6^. Each measurement point consisted of 10 min of stabilization time, followed by 10 min of measurement. Control experiments were conducted in the same setting with the Tyrode solution alone and tied to individual experiment groups based on the time the treatment was given to the cells. Therefore, some control groups can repeat thought-out results. Control EBs were taken from each batch to account for variances among batches. During all experiments, the temperature was maintained at 37 °C.

The resulting data were processed by a python-based script, which located R and S peaks for all contractive events^23^. Contraction forces (nN), beat rates (bpm), and interbeat intervals (sec) were calculated and statistically evaluated. Representative AFM records of vertical deflection of cells treated with salbutamol, aminophylline, and a mix of both are shown in Figure S5.

### Beat rate variability (BRV) analysis

To ensure reliable and valid data, samples invalid for BRV analysis because of multiple sudden irregular pauses in beat rate were excluded using SignalPlant software (ver. 1.2.8.2)^24^. Resulting interbeat RR-intervals were analyzed by domain BRV analysis methods, which compute variability. For analysis of short-term variability, the standard deviation of successive differences (SDSD) was used^25^. SDSD serves as an index of beat-to-beat variability, primarily determined by external influences on the heart. It measures the variability in the time intervals between successive heartbeats. It is unaffected by linear trends in the heart rate, acting as a statistical filter in which the low-frequency components are removed^26^. In our approximation, R-R intervals that deviated from the median by more than two times were excluded from the analysis to reduce the influence of noise. The PyHRV^27^ library for Python was used for SDSD calculations. Internal variability was constant during the measurement and may be related to the mixed population of all three CM subtypes as described elsewhere; in brief, nodal-like CMs had an AP duration at 90% of repolarization shorter than 100 ms (about 16%), slightly more frequent were atrial-like cells, and recorded AP mainly demonstrated a typical ventricular-like shape^28^.

### Statistical analysis

Data was processed via the in-house Python-based scripts or Signal plant software as described above. Statistical evaluation was calculated with GraphPad Prism 8.5 software (GraphPad Software). All data were tested for outliers by the ROUT (Q = 1%) method, and their normal distribution was tested via available normality tests. Ordinary one-way ANOVA with the Holm Sidak multiple comparisons test was used to test the statistical significance of the differences in normally distributed group pairs. In cases where equal standard deviations could not be assumed, Brown–Forsythe ANOVA with Games–Howell multiple comparisons test was used. Where normality tests failed, non-parametric tests were used. Cut-off arrhythmia analysis (Figure S2 and S3, Supplementary data) was previously described^6^. Briefly, in that work, inter-beat RR intervals over 3 s were subtracted, and the statistical significance of the differences between treatments and controls were tested by the Chi-square test with Yate’s correction. An individual statistical test is specified in appropriate figure legends and in Supplementary Data.

## Results

### Inotropic and anti-arrhythmic effect of salbutamol on hPSC-CMs

**Fig. 1:**
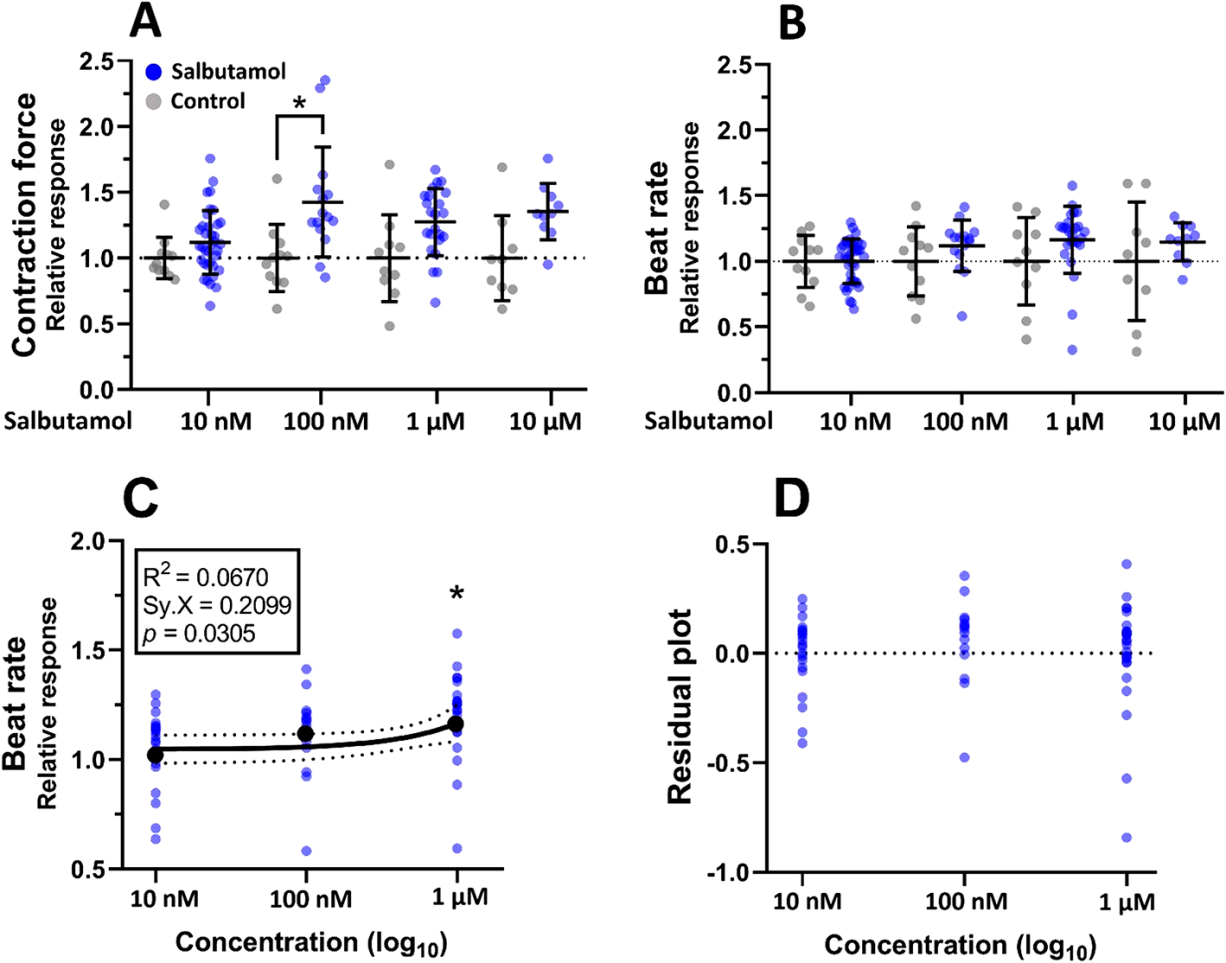
Effect of salbutamol on contraction properties of hPSC-CMs. Scatter plots with indicated means and standard deviation (SD) of the contraction force and beat rate relative responses normalized to the respective baseline control measurement in the case of salbutamol and to mean values of the control measurement in the case of control (*n* = 11 for controls, *n* = 15, 15, 23, and 10 for 10 nM, 100 nM, 1 µM, and 10 µM salbutamol, respectively). At least four biological repetitions were used in each column. **(A)** The contraction force was significantly increased after administration of salbutamol at the concentration of 100 nM (Kruskal-Wallis test with Dunn’s multiple comparisons test, *P* < 0.05). **(B)** The beat rate wasn’t significantly elevated after administration of salbutamol (*P* > 0.05). **(C**,** D)** Linear regression of beat rate relative responses and measured concentrations coupled with residual plot. Results show a statistically significant non-zero slope (Wald test, *P* = 0.0305), proving a positive relationship between concentration and relative response.

The AFM-based setup was used to obtain and calculate the contraction force and beat rate of hPSC-CMs in EBs. The contraction force was significantly increased by 40% in the presence of 100 nM salbutamol compared to the normalized control (Fig. 1A) and increased non-significantly in higher concentrations. Salbutamol didn’t significantly increase the beat rate in the concentration range between 10 nM and 10 µM (Fig. 1B). Still, linear regression of beat rate relative responses and measured concentrations showed a significant increase towards 1 µM (Fig. 1CD).

**Fig. 2:**
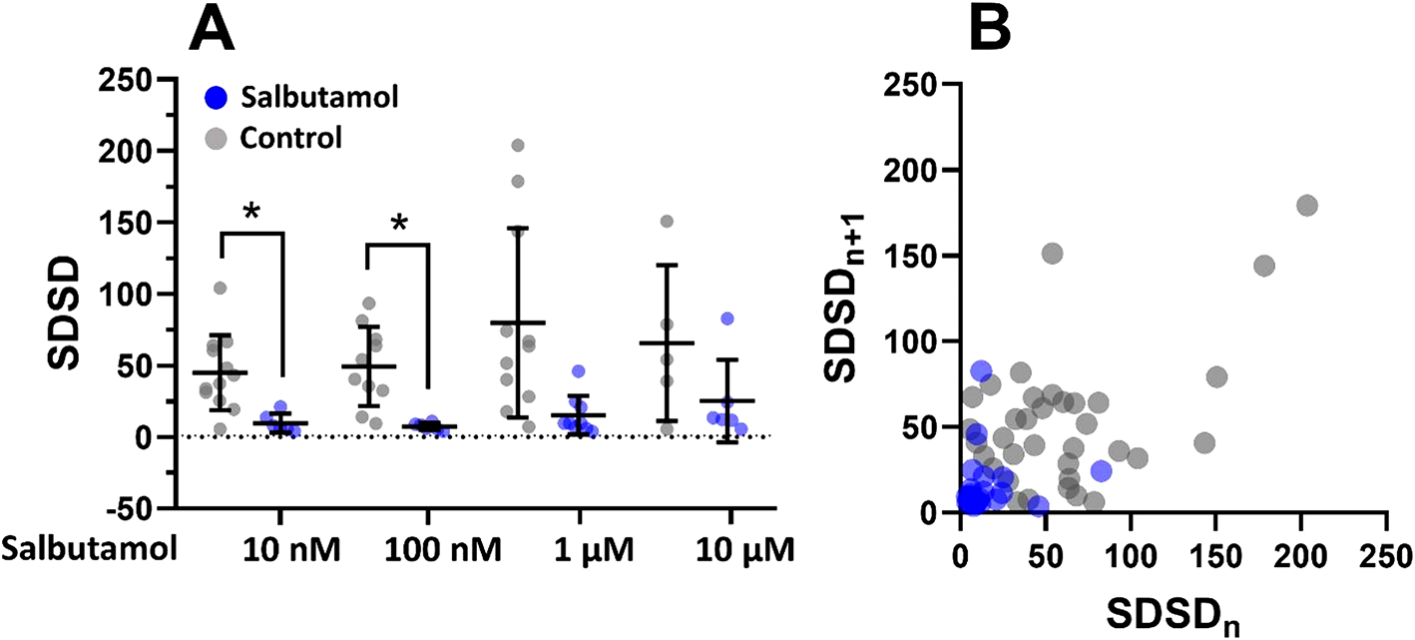
Beat rate variability analysis of hPSC-CMs with salbutamol. **(A)** Scatters plots with indicated means and SD of the standard deviation of successive differences (SDSD) of EBs measured in salbutamol and control (*n* = 8, 9, 9, and 6 for 10 nM, 100 nM, 1 µM, and 10 µM salbutamol, respectively, and *n* = 14, 11, 11, and 5 for the respective controls). SDSD with 10 nM and 100 nM salbutamol were significantly lower compared to controls (Brown-Forsythe and Welch ANOVA test with Holm-Sidak multiple comparisons test, control vs. 10 nM salbutamol, *P* = 0.0115, control vs. 100 nM salbutamol, *P* = 0.0139). **(B)** Poincaré plots with SDSD intervals.

The SDSD of short-term beat rate variability was used for the analysis of arrhythmic effects after salbutamol administration. A higher value corresponds to an elevated beat rate variability, i.e., to an increased pro-arrhythmic activity. Interestingly, salbutamol showed a dose-independent trend to reduce the variability of RR intervals compared to controls (Fig. 2A). This effect is further illustrated in the Poincaré plot, where measurements with salbutamol are clustered in the left bottom corner of the plot (i.e., showing a low variability), compared to the scattered control values (Fig. 2B).

We observed a limited effect of salbutamol on the beat rate and contraction force, with the latter significantly increased at 100 nM salbutamol. While a significant anti-arrhythmic effect was present within all tested concentrations (i.e. 10 nM and 100 nM) of salbutamol.

### Synergic effect of salbutamol and aminophylline on hPSC-CMs

**Fig. 3:**
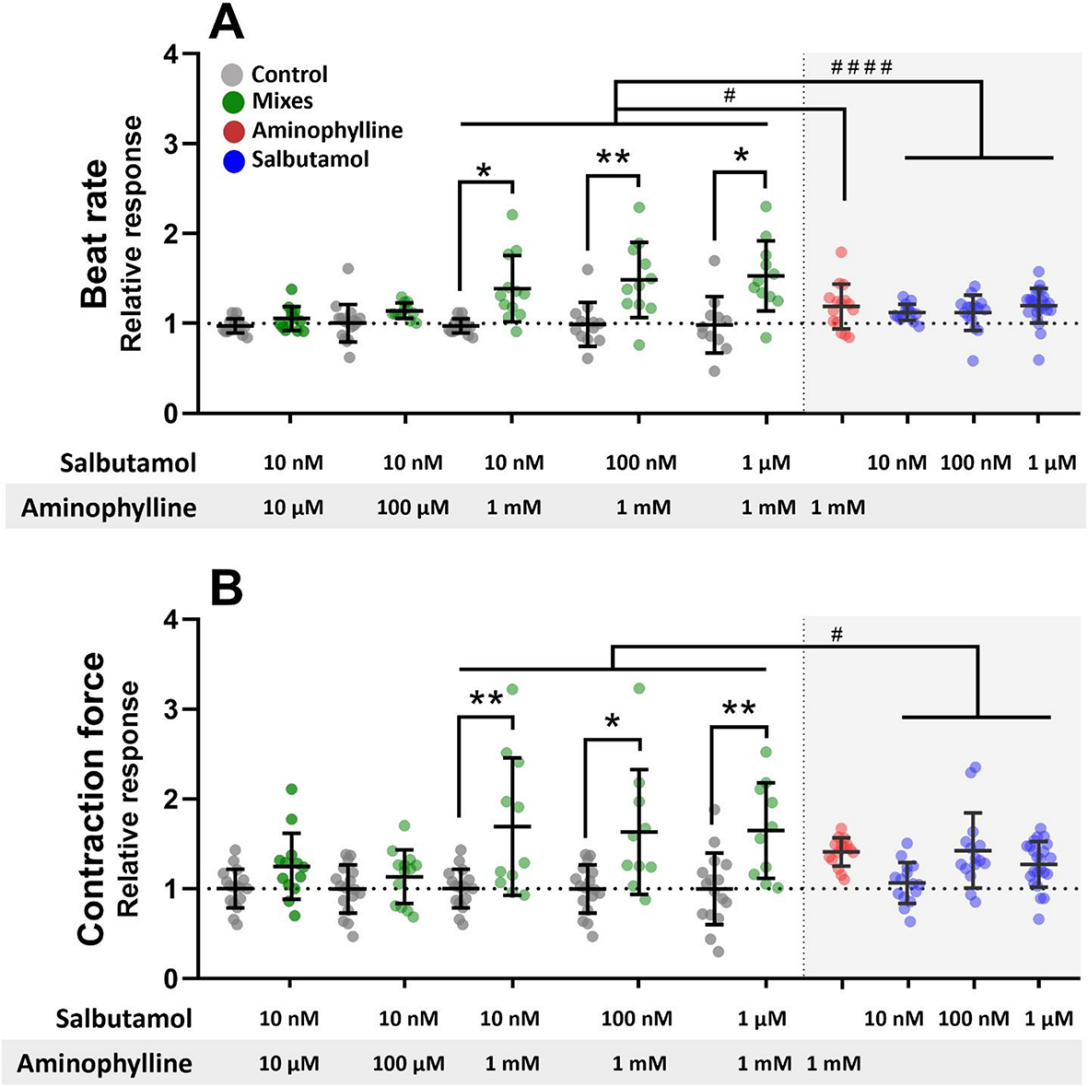
Synergic effect of salbutamol and aminophylline on contractile properties of hPSC-CMs. *stands for *P* ≤ 0.05, **P ≤ 0.01, compared to control, # stands for *P* ≤ 0.05 and #### stands for *P* ≤ 0.0001. Scatter plots with indicated means and standard deviation (SD) of the beat rate (**A**) and contraction force (**B**) relative responses normalized to the respective baseline control measurement in the case of salbutamol and aminophylline and to mean values of the control measurement in the case of control (*n* = 17 for controls, *n* = 13, 13, 11, 10, 10, 15, 15, 23, and 15 for treatments, P values in Table S1 in Supplementary data, Brown-Forsythe and Welch ANOVA test was used for contraction force results, ordinary one-way ANOVA for the beat rate results and Kruskal-Wallis test was applied to compare contraction force and beat rate results of mixes with individual treatment). The legend in A applies to the whole graph. The results of the mixes consist of two separate experiments, same control dataset used based on time of measurement (the first two controls are the same as the third and fourth, relating to 20 min and 40 min of treatment). The results of separate aminophylline administration originate from our previous paper^6^. (**A**,** B)** The beat rate and contraction force significantly differed from controls in mixed treatments with 1 mM aminophylline and 10 nM, 100 nM, or 1 µM salbutamol. Still, no significant effect was observed in individual treatments. The effect of mixed treatments was amplified when compared to individual treatments.

Effects of aminophylline and salbutamol alone were compared with their combined action in various concentrations on EBs prepared from hPSC-CMs derived from the CCTL14 cell line. Significant increases in the beat rate and contraction force were observed when the combinations of 10 nM, 100 nM, and 1 µM salbutamol with 1 mM aminophylline were administered (Fig. 3). Strikingly, a comparison with individual effects of salbutamol and aminophylline in the respective concentrations revealed an amplified effect of the combined drugs. The observed effects on the beat rate and contraction force were also validated using a different cell line (CCTL12 hPSC-CMs; Figure S1 in Supplementary data).

**Fig. 4:**
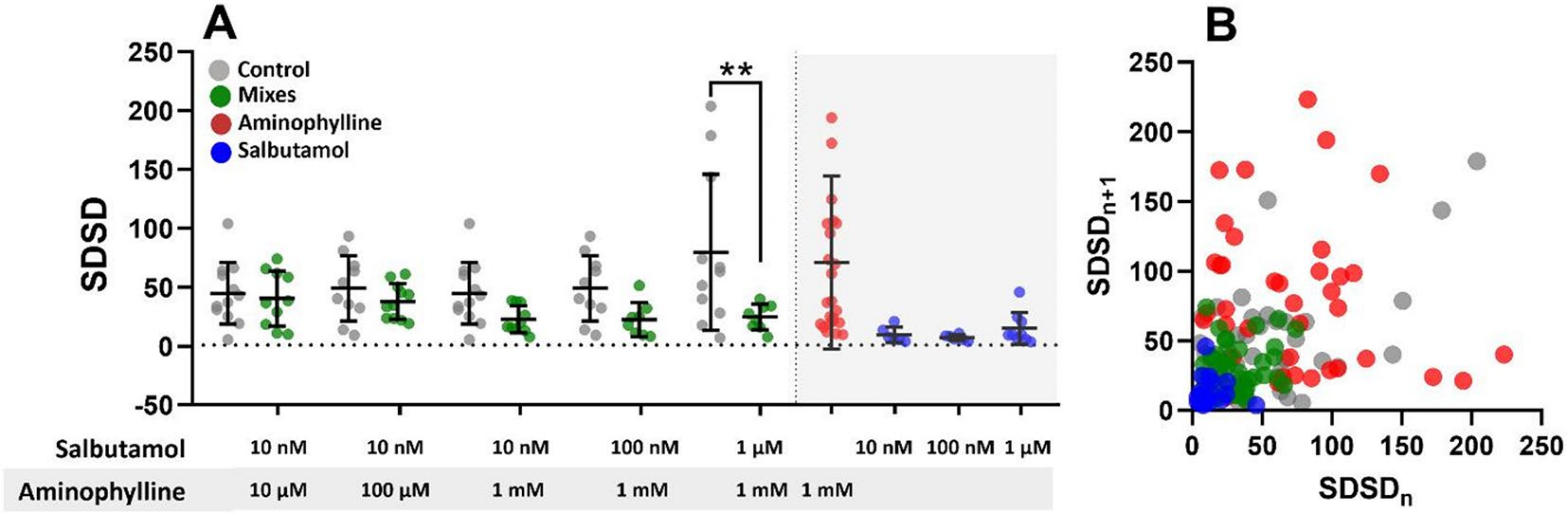
Beat rate variability analysis with combined salbutamol and aminophylline treatments. Scatter plots indicate means and SDs of SDSDs measured with mixed treatments and control (*n* = 10 for controls, *n* = 10, 11, 10, 8, 8, 24, 6, 6, and 9 for treatments, p values in Table S1 in Supplementary data, Ordinary one way ANOVA test was used). **(A)** SDSD results of EBs with indicated concentrations of salbutamol and aminophylline supplemented with separate measurements of both treatments for comparison. The results of the mixes consist of two separate experiments, so the same control dataset was used based on time of measurement (the first two controls are the same as the third and fourth, relating to 20 min and 40 min of treatment). The results of separate aminophylline administration originate from our previous paper^6^. **(B)** Poincaré plots with SDSD intervals.

Analysis of the short-term beat rate variability with combined salbutamol and aminophylline treatment showed a decreasing arrhythmia tendency across the concentrations, with a significant decrease if 1 µM salbutamol and 1 mM aminophylline were applied (Fig. 4A, B). In our previous study, we demonstrated the arrhythmogenic potential of aminophylline using the cut-off method of arrhythmia analysis^6^. An analogical cut-off arrhythmia analysis of the current data revealed an anti-arrhythmic effect of salbutamol, alone or administered with aminophylline (Figure S2 and S3).

Overall, we observed a synergic effect of salbutamol and aminophylline as both bronchodilators potentiate their individual effects on the contraction force and beat rate; this effect was most apparent with 1 mM concentration of aminophylline. Finally, anti-arrhythmic effect of salbutamol prevailed even aminophylline was co-administered.

### Activation of nitric oxide synthase (NOS) reduces aminophylline induced arrhythmia

**Fig. 5:**
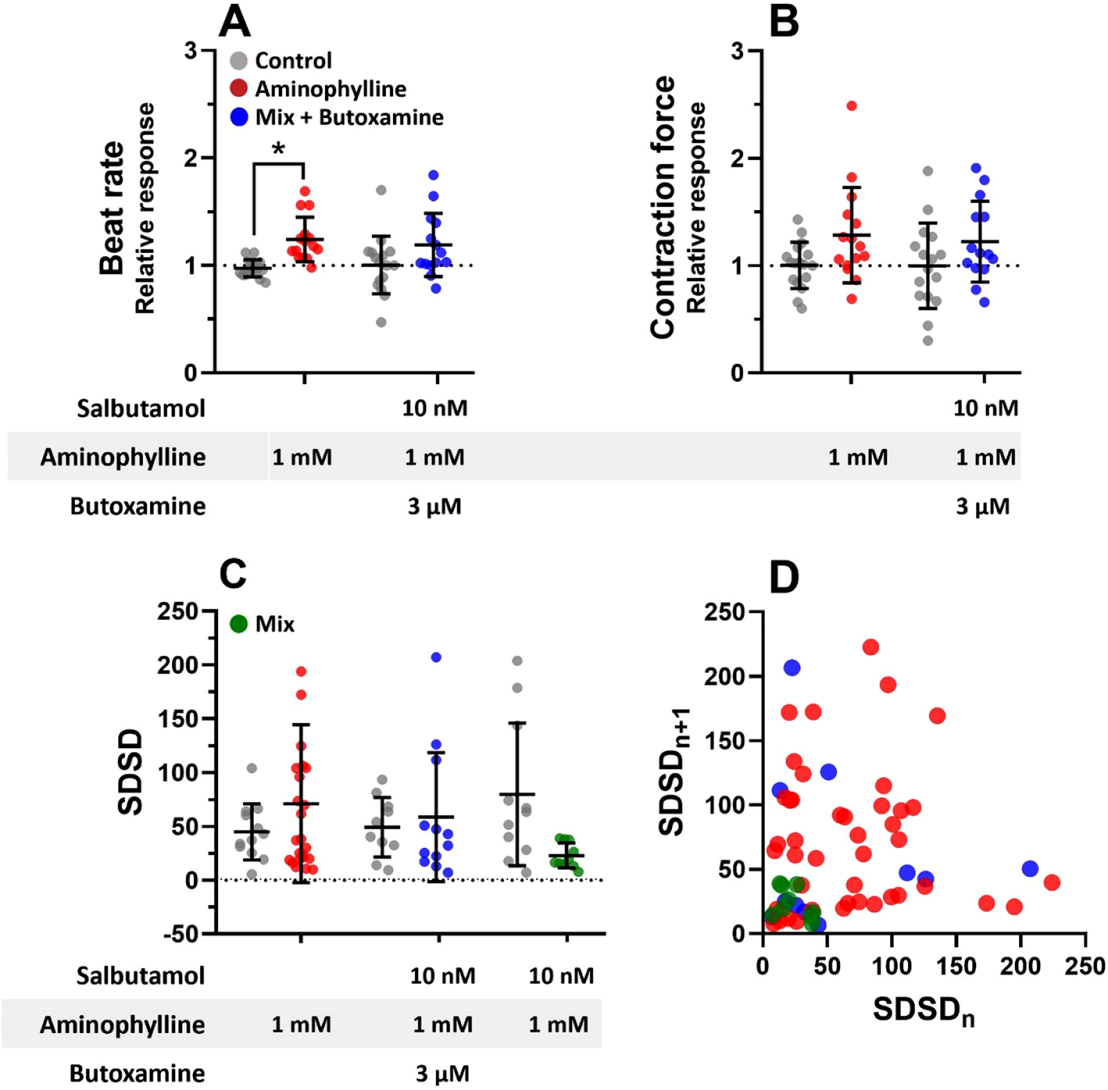
Contractile properties and beat rate variability analysis of hPSC-CMs with salbutamol, aminophylline, and selective β_2_-AR blocker butoxamine. Scatter plots indicate means and standard deviations (SD) of the beat rate and contraction force relative responses normalized to the respective baseline control measurement in the case of salbutamol and mixed treatments and to mean values of the control measurement. Scatter plots with means and SDs of SDSDs measured with mixed treatments and control means (n and P values) are presented in Table S1 in Supplementary data, ordinary one-way ANOVA test was used. **(A**,** B)** The contraction force and beat rate of EBs treated with the mixes of salbutamol, aminophylline, and butoxamine are indicated at the bottom. **(C)** Analysis of short-term variability SDSD. Control groups were always paired with treatment groups based on the time they were given, so they were repeated from previous figures. **(D)** Poincaré plots with SDSD. If butoxamine was added to the mix, contractile properties were not significantly different from the effect of aminophylline alone. The anti-arrhythmic potential of salbutamol completely diminished when butoxamine was co-administered.

To exclude a contribution of possible non-specific mechanisms of the salbutamol action, we investigated the role of beta-2 adrenergic receptors (β_2_-AR) in the observed effect. We co-administered butoxamine, a β2-selective blocker, with the mixture of salbutamol and aminophylline, and we observed that butoxamine effectively blocked the anti-arrhythmic effects of salbutamol and synergic effects of the salbutamol-aminophylline combination (Fig. 5). Interestingly, when treatment containing salbutamol was administered before any treatment containing butoxamine, the anti-arrhythmic effect was apparent even after the addition of pro-arrhythmic aminophylline alone. (Figure S5 in Supplementary data). These results showed that the anti-arrhythmic effect in hPSC-CM is mediated solely through β_2_-AR and could last tens of minutes after salbutamol exposure.

**Fig. 6:**
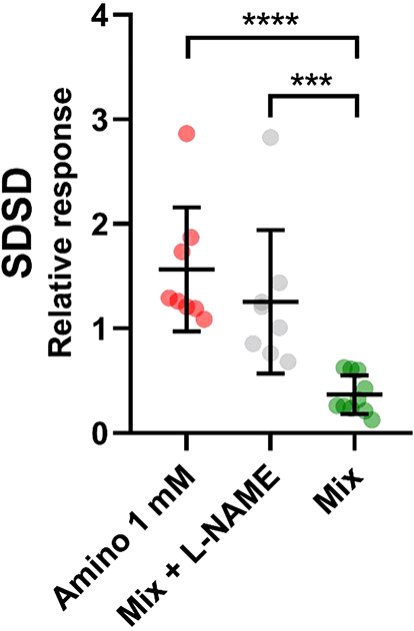
Beat rate variability analysis of hPSC-CMs with salbutamol, aminophylline, and L-NAME, non-selective inhibitor of NOS. Figure shows relative responses to the respective baseline control. Results shows that L-NAME is co-administrated with mix of salbutamol (10 nM) and aminophylline (1 mM), SDSD values are comparable to results with aminophylline (1mM) and significantly increased to salbutamol and aminophylline mix without the inhibitor (Amino 1 mM vs. Mix, *p* = 0.0001, Mix + L-NAME vs. Mix, *p* = 0.0065, Kruskal-Wallis with Dunn’s multiple comparisons test, *n* = 8 for Amino 1 mM and Mix + L-NAME, *n* = 10 for Mix).

To test whether β_2_-AR activated NOS has a role in antiarrhythmic effect of salbutamol, a well-known non-specific inhibitor of NOS Nω-Nitro-L-arginine methyl (L-NAME) was co-administered with mixture of salbutamol and aminophylline. Figure 6 shows that inhibition of NOS led to significant increase of SDSD value compared to mix without the inhibitor. This increase was comparable to results where sole aminophylline was administered (Fig. 6, *P* > 0.01). These results indicate that mechanism of salbutamol anti-arrhythmic effect depends on activation NOS through β_2_-AR.

## Discussion

In the present study, we observed that salbutamol at clinically relevant concentrations up to 1 µM, which should selectively affect β_2_-AR, caused a significant inotropic effect and a non-significant trend toward a chronotropic effect on hPSC-CM clusters. When salbutamol was applied with aminophylline, inotropic and chronotropic effects were more prominent. Importantly, this is the first report on a significant anti-arrhythmic effect of salbutamol, which reduced the arrhythmia occurrence previously introduced with aminophylline in our hPSC-CMs model. This anti-arrhythmic effect was blocked by a selective β_2_-AR blocker butoxamine and by a non-selective NOS inhibitor L-NAME.

The positive inotropic effect and the concentration-dependent trend to a positive chronotropic effect of salbutamol are consistent with its known stimulatory effect on β_2_-AR^29–32^. This receptor is primarily expressed in cells of the lower respiratory tract, such as in smooth muscle cells, where its activation leads to relaxation and, thus, to bronchodilation^33^. In cardiac cells, there is approximately a 4:1 ratio of β_1_-AR to β_2_-AR; activation of both predominantly leads to chronotropy, inotropy, and lusitropy^34,35^. Similarly, we found lower expression of β_2_ receptors compared to β_1_ in our model (described in our previous work^36^). These effects are mediated by the activation of protein kinase A, which phosphorylates several effector molecules such as phospholamban (PLB), L-type calcium channels (LTCC), or troponin complex^37,38^ which plays a key role in the regulation of inotropy ^39^. Phosphorylation of troponin during activation of β-adrenergic receptors causes a more intense response even at lower Ca^2+^ concentrations^39,40^. Stimulation of β_1_-AR, but not that of β_2_-AR, may show proarrhythmic effects^41^, and as suggested, β_2_-AR receptors may even counteract β_1_-AR mediated arrhythmogenic effect^42^. The minimal but significant chronotropic effect of salbutamol is not surprising considering its β_2_ selectivity and preferential activity in the bronchial smooth muscle^29,32^ as well as much lower expression of β_2_ receptors in healthy myocardium compared with β_1_ receptors^35^. The chronotropic effect of isoproterenol, a non-selective β-AR agonist, is much higher. It was shown that salbutamol at concentrations below 1 µM (which primarily stimulate β_2_ receptors and were used in our study) did not cause a chronotropic effect, and its chronotropic effect at higher concentrations was mediated exclusively through β_1_ receptor stimulation^43^.

As confirmed in this study (with the use of butoxamine), the observed anti-arrhythmic effect of salbutamol was primarily mediated *via* the β_2_-AR. The chronotropic effect was smaller than that of isoproterenol, which corresponds to clinical experience^44,45^. Nevertheless intrinsic beat rate variability of the model may contribute to the observed trend.

In patients with obstructive lung diseases, various combinations of bronchodilator drugs are used to relieve symptoms^1–3^. While the side effects of individual bronchodilator drugs on cardiac physiology were studied, the side effects of combinations of these substances have not been clarified sufficiently^46–48^. Hence, we decided to analyze the combined effect of different concentrations of salbutamol and aminophylline. We discovered that the combination of salbutamol and aminophylline in concentrations of at least 10 nM and 1 mM had synergic chronotropic and inotropic effects. These effects were significantly stronger compared to controls but also to individual measurements with aminophylline or salbutamol. Aminophylline, similar to other methylxanthines such as caffeine, nonspecifically inhibits phosphodiesterase (PDE) and antagonizes adenosine receptors^49^. Taken together, salbutamol (by activation of β_2_-AR) and aminophylline (by inhibition of PDE and adenosine receptors) co-medication may result in an increased intracellular concentration of cAMP and synergic chronotropic and inotropic effects.

Surprisingly, the pro-arrhythmic effect of aminophylline described in our previous study ^6^ was reduced in the presence of salbutamol. The previously proposed mechanism of the aminophylline arrhythmic effect was connected to a microdomain cAMP level-related diastolic Ca^2+^ releases *via* independent clusters of ryanodine receptor 2 (RyR2) channels, reducing the threshold for spontaneous Ca^2+^ release^50^. Since the antiarrhythmic action of salbutamol, both alone and in the presence of aminophylline, cannot be logically mediated by the cAMP-PKA pathway, which is generally known to show an opposite effect^51–54^. We were looking for alternative possibilities. Considering the role of the cGMP-eNOS pathway, which may be activated by β2-AR^55–60^ and is known to cause a systemic decrease of cytosolic Ca^2+^ concentration and increase in SR load^57–60^, thus likely inhibiting spontaneous Ca^2+^ release from SR, we aimed to prove if the non-specific NOS inhibitor L-NAME could mitigate or even prevent the antiarrhythmic effect of salbutamol. The cGMP pathway also depends on cAMP level^55,61^. As apparent from Fig. 6, SDSD was not different if both salbutamol and aminophylline were applied in the presence of L-NAME from SDSD in the presence of aminophylline alone and, vice versa, it was significantly higher to SDSD in the presence of salbutamol and aminophylline mixture, thus, proving the central role of cGMP-eNOS pathway in the antiarrhythmic action of salbutamol.

Our main finding showing that salbutamol reduces the arrhythmogenic effect of aminophylline may be highly clinically relevant. The arrhythmogenic effect of aminophylline is widely known, occurring in about 13% of patients after intravenous administration of the drug or even in 20% of overdosed patients^5,9^. Aminophylline-induced arrhythmia presents most frequently as atrial fibrillation, but more severe arrhythmias may also occur^62^. The dual treatment with salbutamol and aminophylline is a frequently used treatment combination in clinical practice, mainly in the hospital setting during acute exacerbations of asthma or COPD^1–3^. No randomized trials have been reported on the combined effect of aminophylline and salbutamol in patients with COPD or asthma. Only sparse data on the potential toxicity of this combination have been reported previously^8^. Our data show two types of results, i.e., that salbutamol added to aminophylline may cease some arrhythmia mechanisms while enhancing the chronotropic and inotropic effects can lead to increased tachycardia, similar to literature^63,64^.

This study first showed that salbutamol at clinically relevant concentrations may reduce the pro-arrhythmic potential of aminophylline if both these drugs are combined. This effect was mediated *via* β_2_-AR activated NOS. The clinical relevance of this finding may be high, representing a potential way to reduce pharmacotoxicity during aminophylline treatment. The combined effect of the two molecules, their safety profile, and potential arrhythmogenicity need further investigation in prospective randomized clinical trials.

## Electronic supplementary material

Below is the link to the electronic supplementary material.

Supplementary Material 1

Supplementary Material 2

Supplementary Material 3

Supplementary Material 4

Supplementary Material 5

Supplementary Material 6

Supplementary Material 7

## Data Availability

Data is provided within the manuscript or supplementary information files.

## Abbreviations

AFM: Atomic force microscopy
BPM: Beats per minute
BRV: Beat rate variability
CBBs: Cell-based biosensors
CCTL: Center for cell therapy line
CM: Cardiomyocyte
EB: Embryoid body
ECC: Excitation-contraction coupling
hESC: Human embryonic stem cell
hiPSC: Human induced pluripotent stem cell
hPSC: Human pluripotent stem cell (hESC and hiPSC)
hPSC-CMs: Pluripotent stem cell-derived cardiomyocytes
SDSD: Standard deviation of successive differences
β_1_-AR: β_1_-adrenergic receptor
β_2_-AR: β_2_-adrenergic receptor
