# Supplementary material for: Salbutamol attenuates arrhythmogenic effect of aminophylline in a hPSC-derived cardiac model
Source: Sci Rep. 2024 Nov 9;14:27399. doi: 10.1038/s41598-024-76846-4 (PMC11550379; doi:10.1038/s41598-024-76846-4)
Supplement: Supplementary file 1 — Supplementary Material 1 [file 41598_2024_76846_MOESM1_ESM.docx]

Supplementary Material

Salbutamol attenuates arrhythmogenic effect of aminophylline in a 3D *in vitro* model.

***Daniil Kabanov^1^, Simon Klimovic^1^, Deborah Beckerova^2^, Martin Molcan^3^, Martin Scurek^4^, Kristian Brat^4^, Marketa Bebarová^5^, Vladimir Rotrekl^2^, Jan Pribyl^6^, Martin Pesl^7^***

*^1^ CEITEC MU, Department of Biochemistry, Faculty of science and Department of Medicine, Faculty of Medicine, Masaryk University, Brno, Czechia*
*^2^ International Clinical Research Center, St. Anne’s University Hospital and Department of Biology, Faculty of Medicine, Masaryk University, Brno, Czechia*
*^3^ Department of Biology, Faculty of Medicine, Masaryk University, Brno, Czechia*
*^4^ Department of Respiratory Diseases, University Hospital Brno and Faculty of Medicine, Masaryk University, Brno, Czechia*
*^5^ Department of Physiology, Faculty of Medicine, Masaryk University, Brno, Czech Republic.*
*^6^ CEITEC MU and Department of Biology, Faculty of Medicine, Masaryk University, Brno, Czechia*
*^7^ International Clinical Research Center, First Department of Internal Medicine—Cardioangiology, St. Anne’s University Hospital and Department of Biology, Faculty of Medicine, Masaryk University, Brno, Czechia*

*** Correspondence:**
Jan Přibyl, Ph.D
jan.pribyl@ceitec.muni.cz Martin Pešl, Ph.D
pesl@fnusa.cz

|  | **Mean Diff.** | **Summary** | **Adjusted P Value** |
| --- | --- | --- | --- |
| **Cumulative effect - contraction force** |  |  |  |
| S. 10 nM+ A. 1 mM vs. ctrl | 0.6895 | ** | 0.0049 |
| S. 100 nM + A. 1 mM vs. ctrl | 0.6335 | * | 0.0117 |
| S. 1 µM+ A. 1 mM vs. ctrl | 0.6499 | * | 0.0117 |
| **Cumulative effect - beat rate** |  |  |  |
| S.10 nM+ A.1 mM vs. ctrl | 0.4152 | * | 0.0105 |
| S.100 nM+ A. 1 mM vs. ctrl | 0.492 | ** | 0.0042 |
| S.1 µM+ A. 1 mM vs. ctrl | 0.5445 | ** | 0.0015 |
| **Cumulative effect - SDSD** |  |  |  |
| S. 1 µM + A. 1 mM vs. ctrl | -54.78 | ** | 0.0076 |
| **Cumulative vs. Individual effect – contraction force** |  |  |  |
| Mixes vs. salbutamol | 17.56 | * | 0.0206 |
| **Cumulative vs. Individual effect – beat rate** |  |  |  |
| Mixes vs. salbutamol | 32.17 | **** | <0.0001 |
| Mixes vs. aminophylline | 19.6 | * | 0.0363 |
| **hPSC-CMs CTTL12 - beat rate** |  |  |  |
| A 1 mM + S 10 nM vs. ctrl | 0.6219 | ** | 0.0012 |
| **hPSC-CMs CTTL12 - contraction force** |  |  |  |
| A 1 mM vs. ctrl | 0.4552 | ** | 0.0073 |
| A 1 mM + S 10 nM vs. ctrl | 0.4933 | ** | 0.0048 |

**Table S1:** Statistical differences calculated for relative responses of contraction force of hPSC-CMs with salbutamol and its mixes with aminophylline and control. *Ordinary one-way ANOVA with Tukey's multiple comparisons test was used in all cases except for Cumulative effect – contraction force, where Brown-Forsythe and Welch ANOVA with Holm-Sidak's multiple comparisons test was used, and Kruskal-Wallis with Dunn’s test was used to compare cumulative and individual effects.*


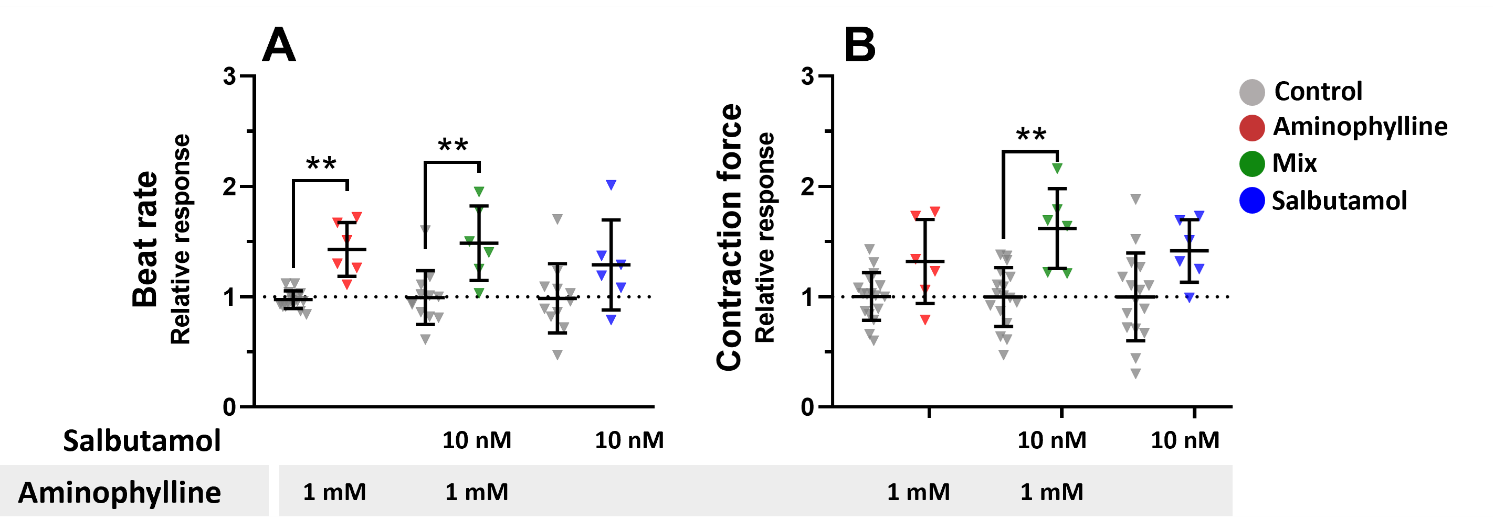


Figure S1 Effect of salbutamol, aminophylline, and its mix on (A) beat rate and (B) contraction force on hPSC-CMs CTTL12 cellular line. *Scatter plots with indicated means and standard deviation (SD) of contraction force and beat rate relative responses normalized to a baseline measurement and to a control measurement means (n = 17 for ctrl, n = 6 for all other treatments, P values are listed in Table S1, one-way ANOVA test was used). At least three biological repetitions were used in each column. Results confirmed effects of salbutamol and aminophylline manifested on CTTL14 cellular line.*


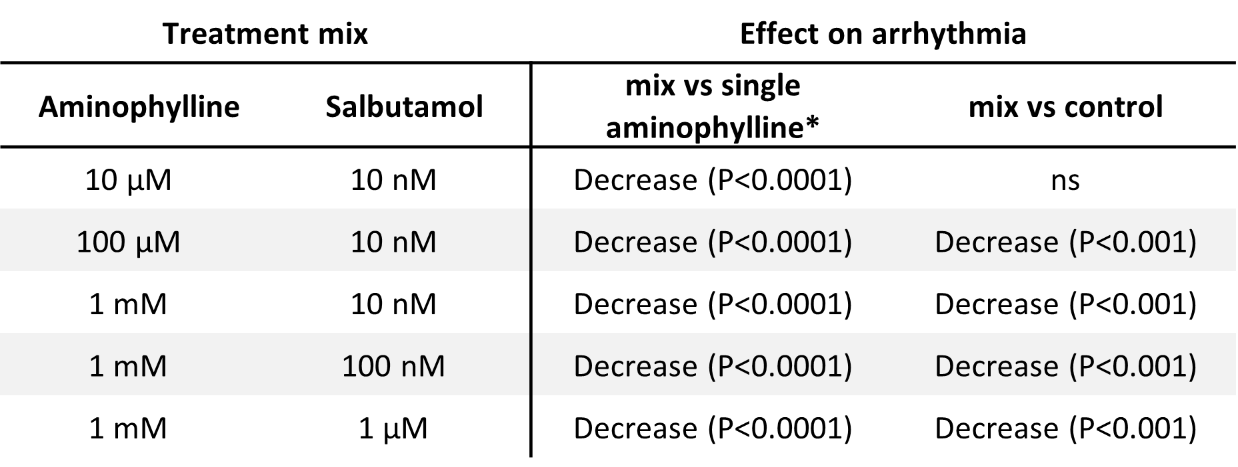


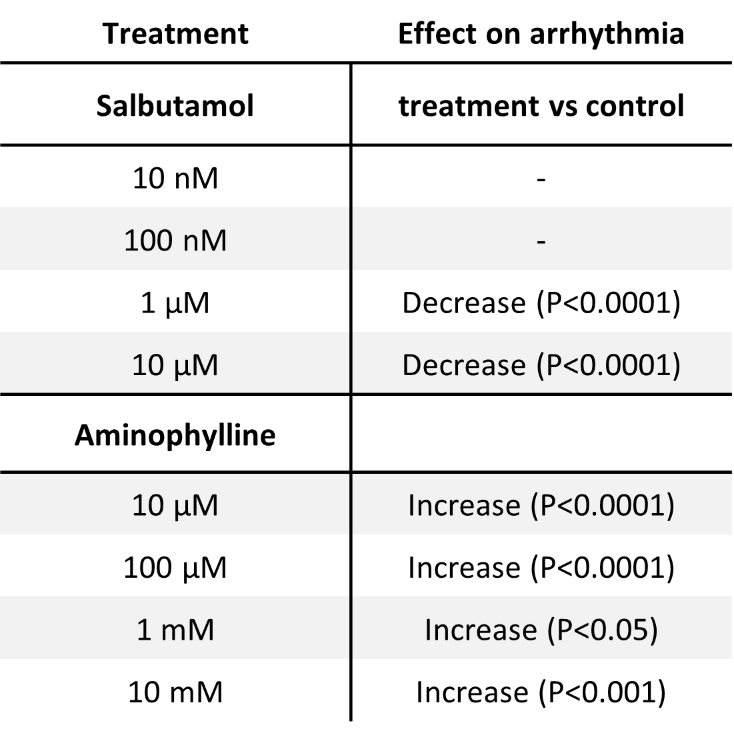
Figure S2: Analysis of arrhythmogenic effect on mixed treatments on hPSC-CMs. *R-R values over 3 second in case of hPSC-CMs measurements and control measurements were subtracted and resulting contingency tables were statistically analyzed with chi-square test with Yates’s correction.*

Figure S3: Analysis of arrhythmogenic effect of salbutamol and aminophylline on hPSC-CMs. *R-R values over 3 second in case of hPSC-CMs measurements and control measurements were subtracted and resulting contingency tables were statistically analyzed with chi-square test with Yates’s correction.*


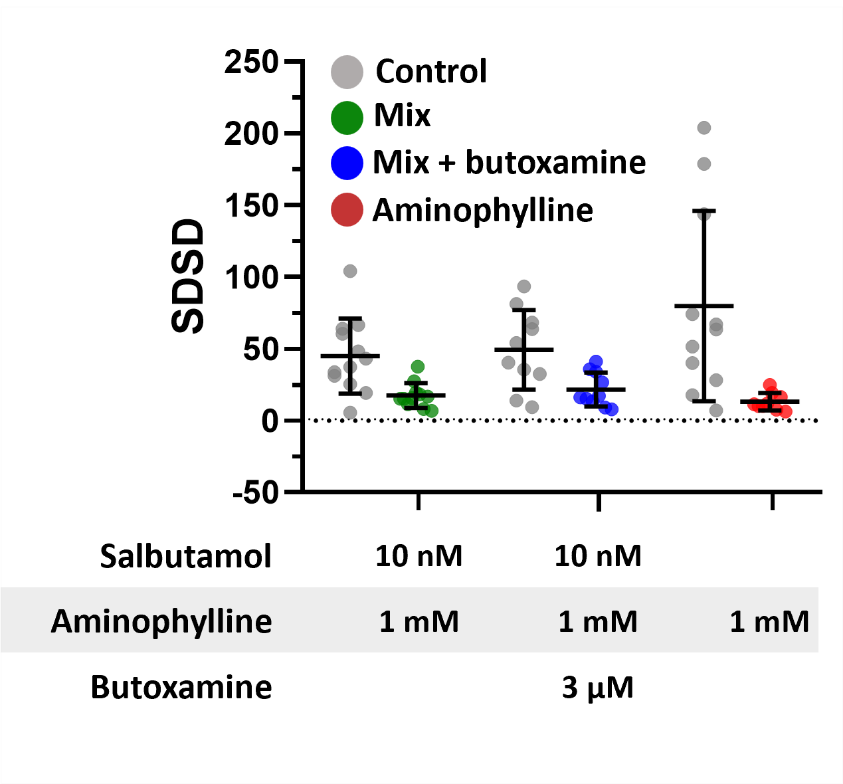
**Figure S4** Beat rate variability analysis of hPSC-CMs with β2-selective beta blocker butoxamine. *Scatters plot with indicated means and SD of and SDSD of EBs measured with mixed treatments and controls means (n = 12, 10 and 11 for controls, n = 11, 10 and 9 for A 1 mM + S 10 nM, A 1 mM + S 10 nM + B 3 µM and A 1 mM respectively). Results showed that when salbutamol was administered in a first treatment, its anti-arrhythmic effect prevail even with addition of butoxamine or when only aminophylline was administered.*

*
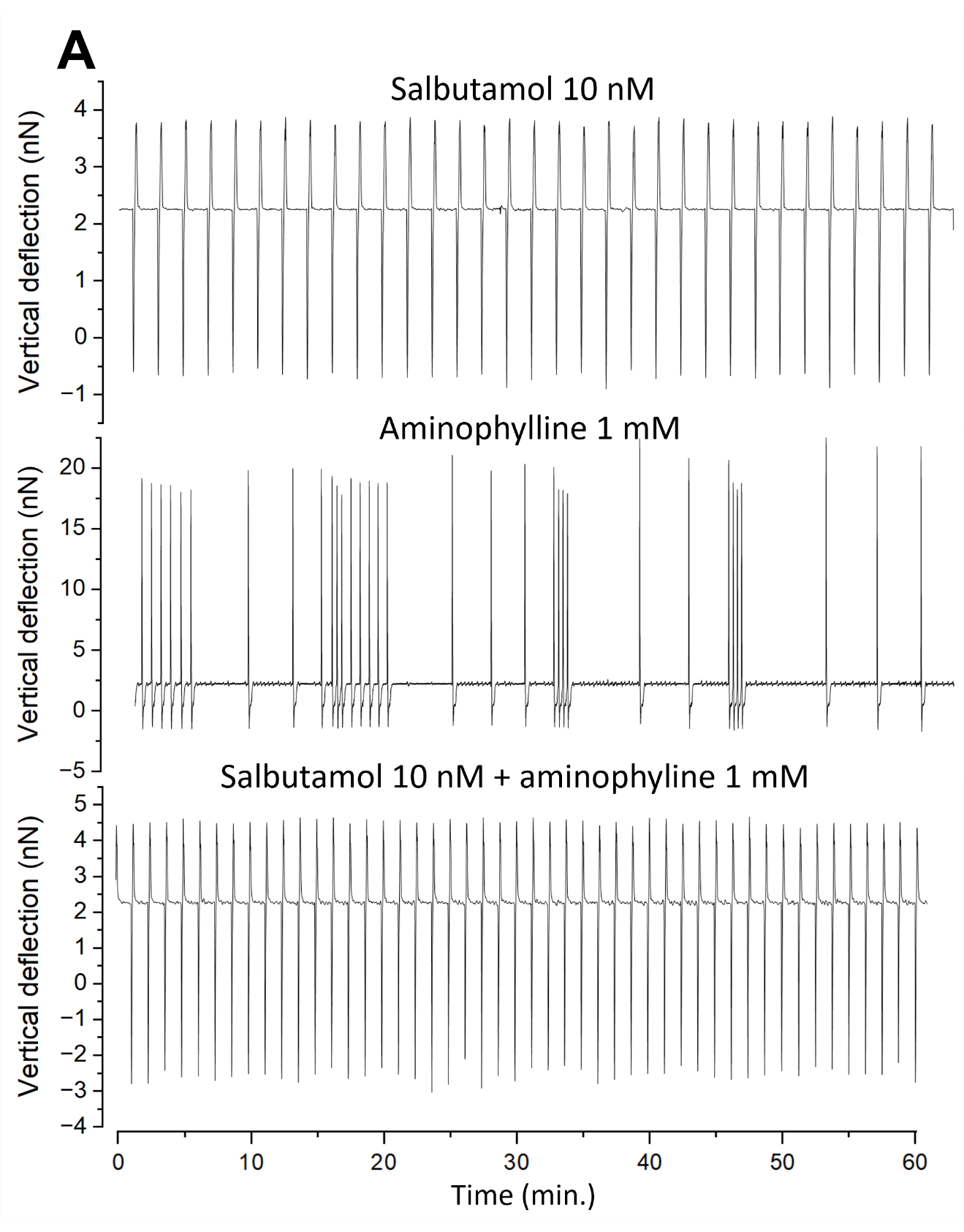
*

**Figure S5** Representative records of vertical deflection measured with hPSC-CMs treated with salbutamol 10nM, aminophylline 1 mM, and mix of both.

*
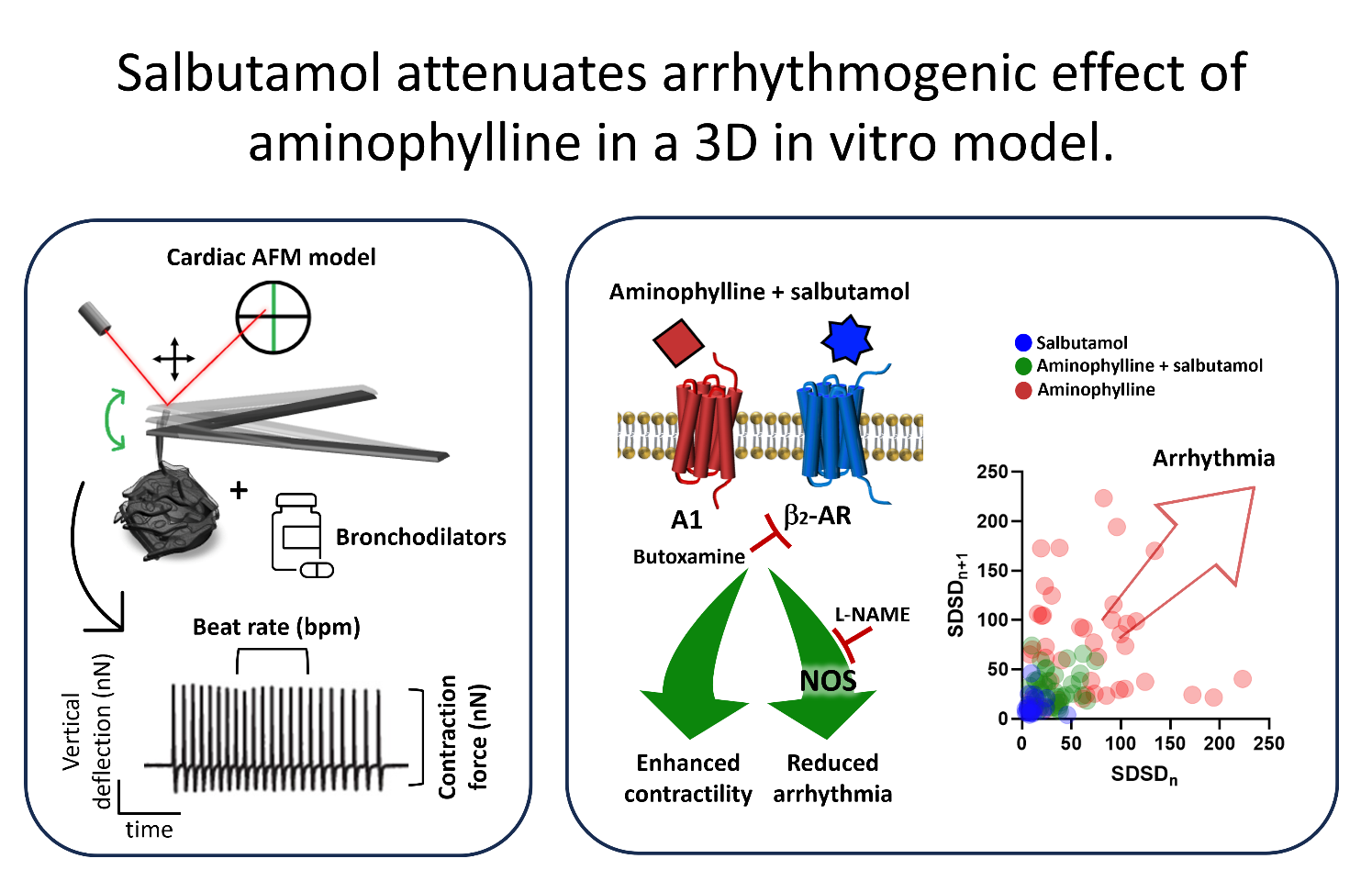
*

**Figure S6 Graphical abstract**
