# Supplementary figures and images for: Salbutamol attenuates arrhythmogenic effect of aminophylline in a hPSC-derived cardiac model
Source: Sci Rep. 2024 Nov 9;14:27399. doi: 10.1038/s41598-024-76846-4 (PMC11550379; doi:10.1038/s41598-024-76846-4)

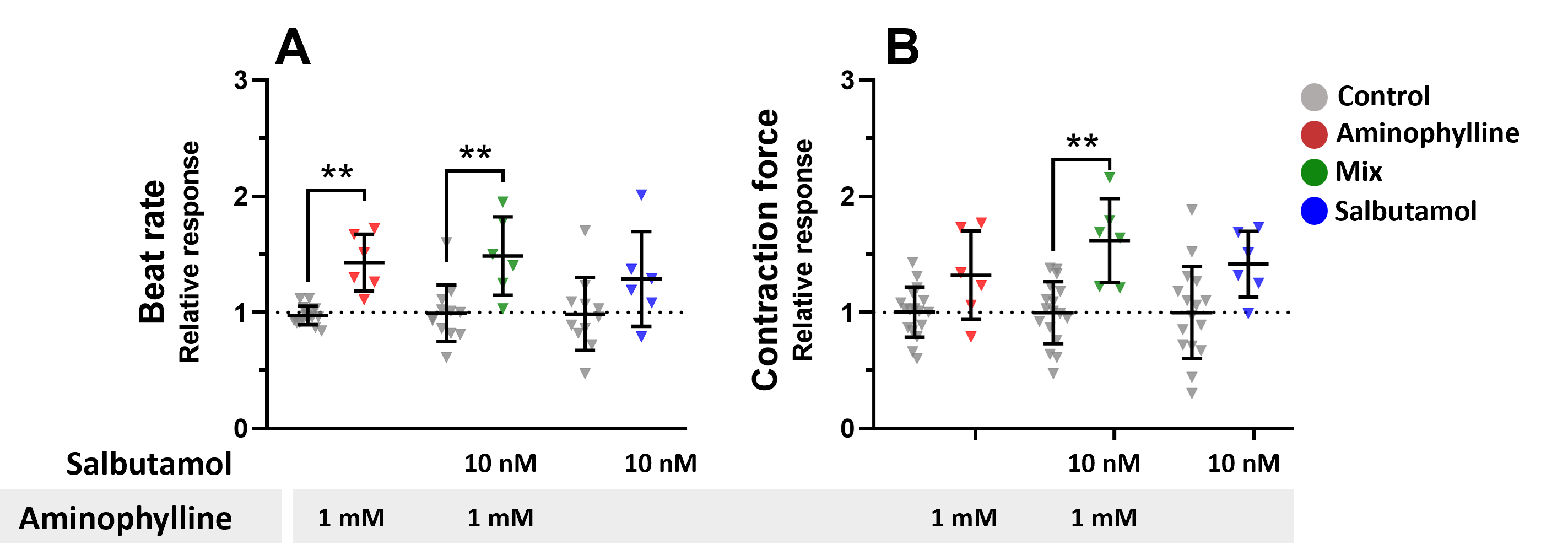

Supplement: Supplementary file 2 — Supplementary Material 2 [file 41598_2024_76846_MOESM2_ESM.tif]

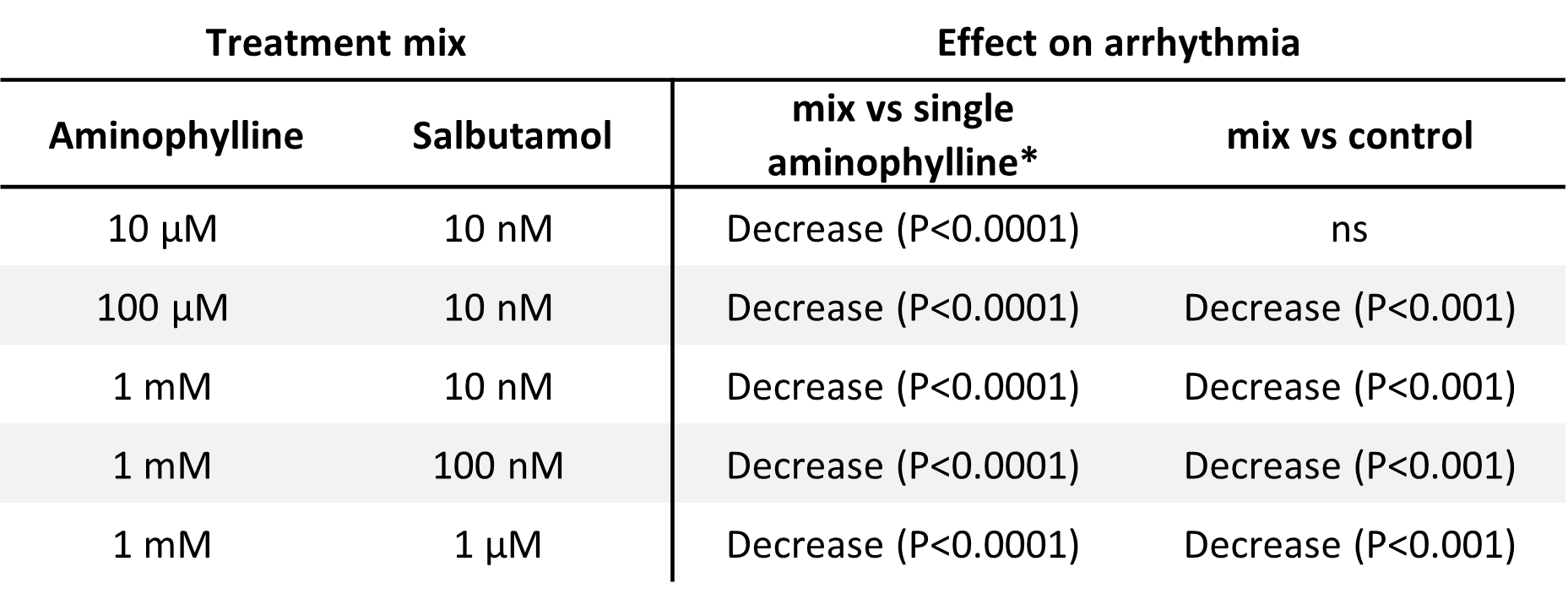

Supplement: Supplementary file 3 — Supplementary Material 3 [file 41598_2024_76846_MOESM3_ESM.tif]

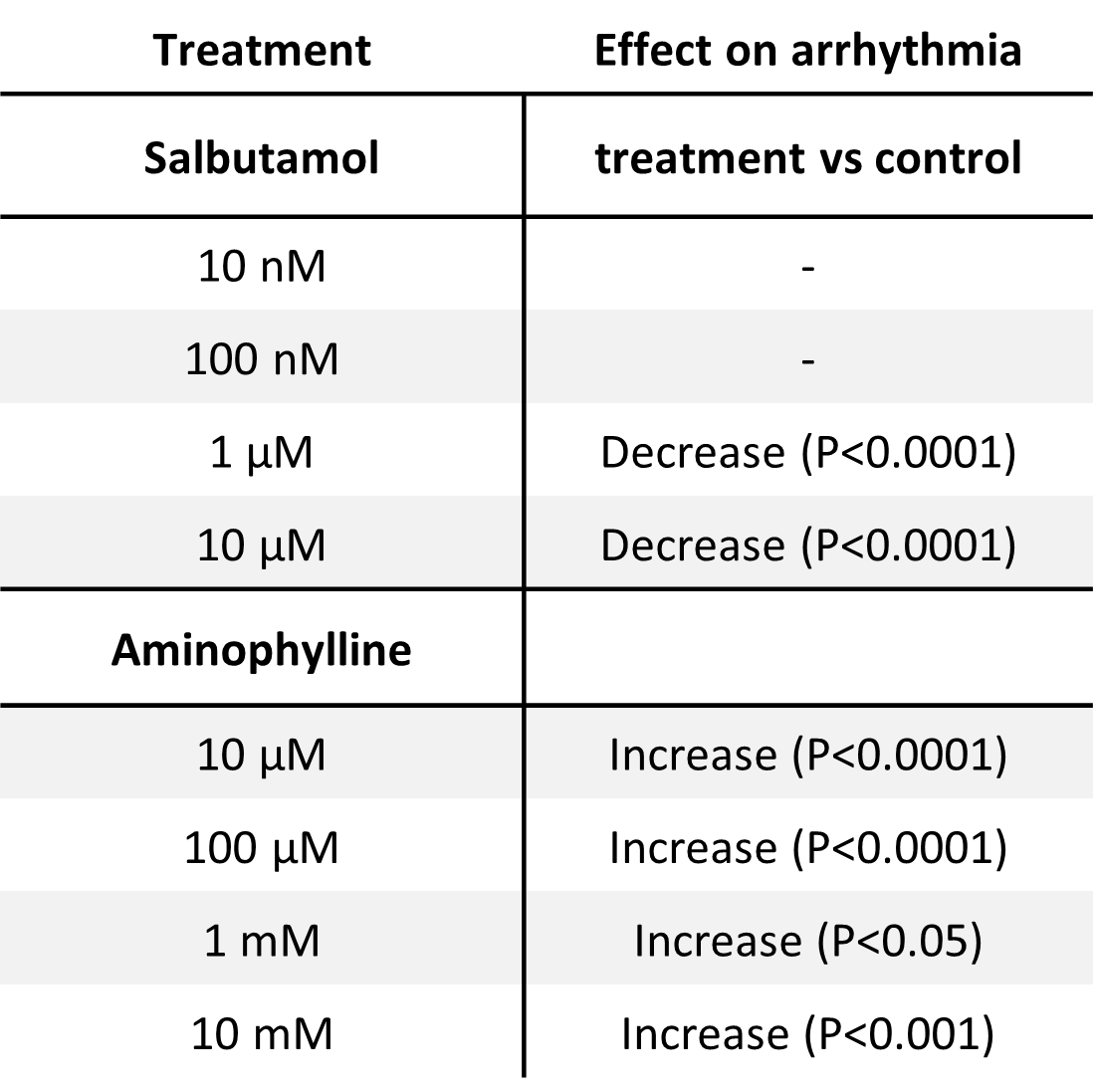

Supplement: Supplementary file 4 — Supplementary Material 4 [file 41598_2024_76846_MOESM4_ESM.tif]

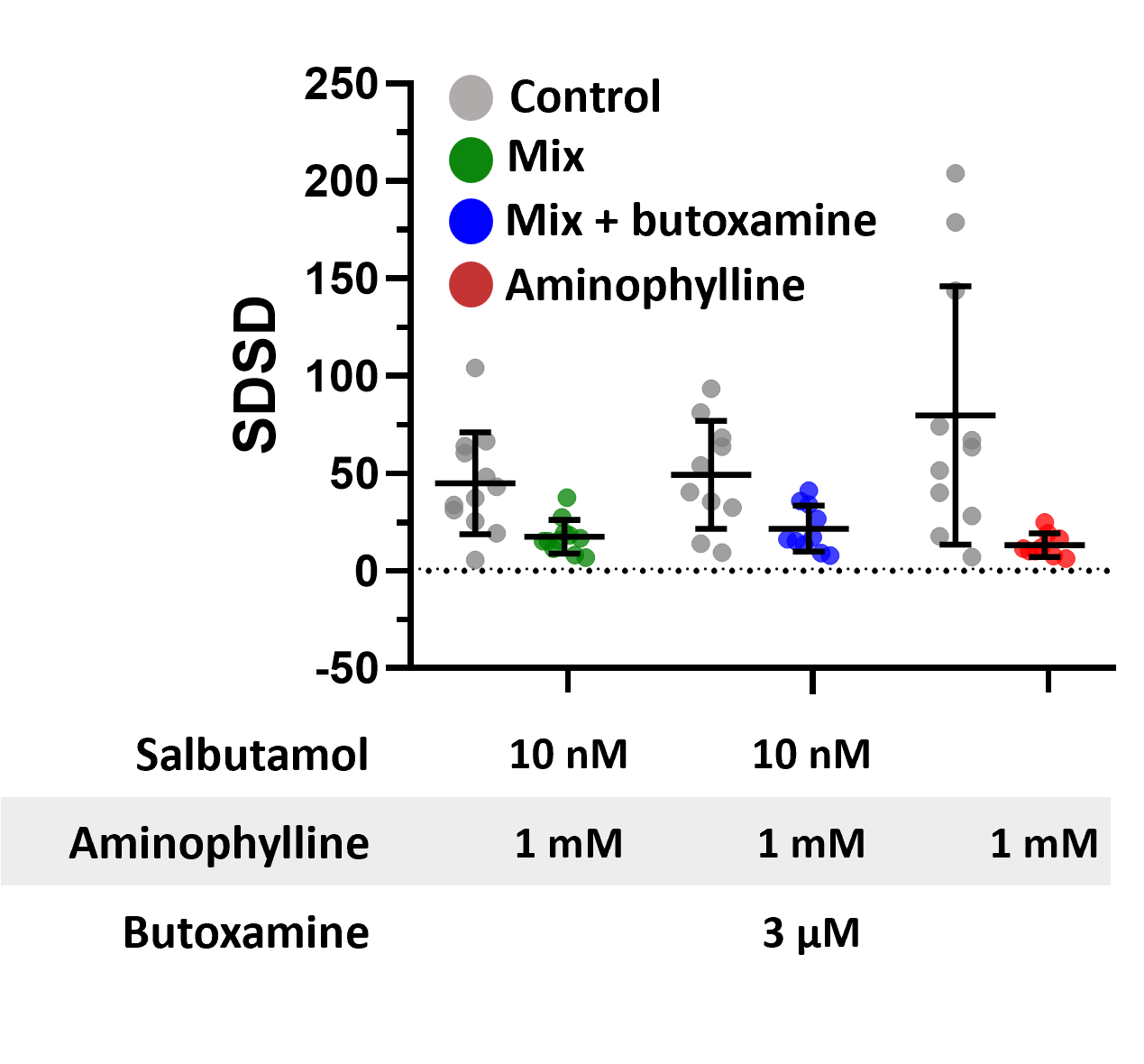

Supplement: Supplementary file 5 — Supplementary Material 5 [file 41598_2024_76846_MOESM5_ESM.tif]

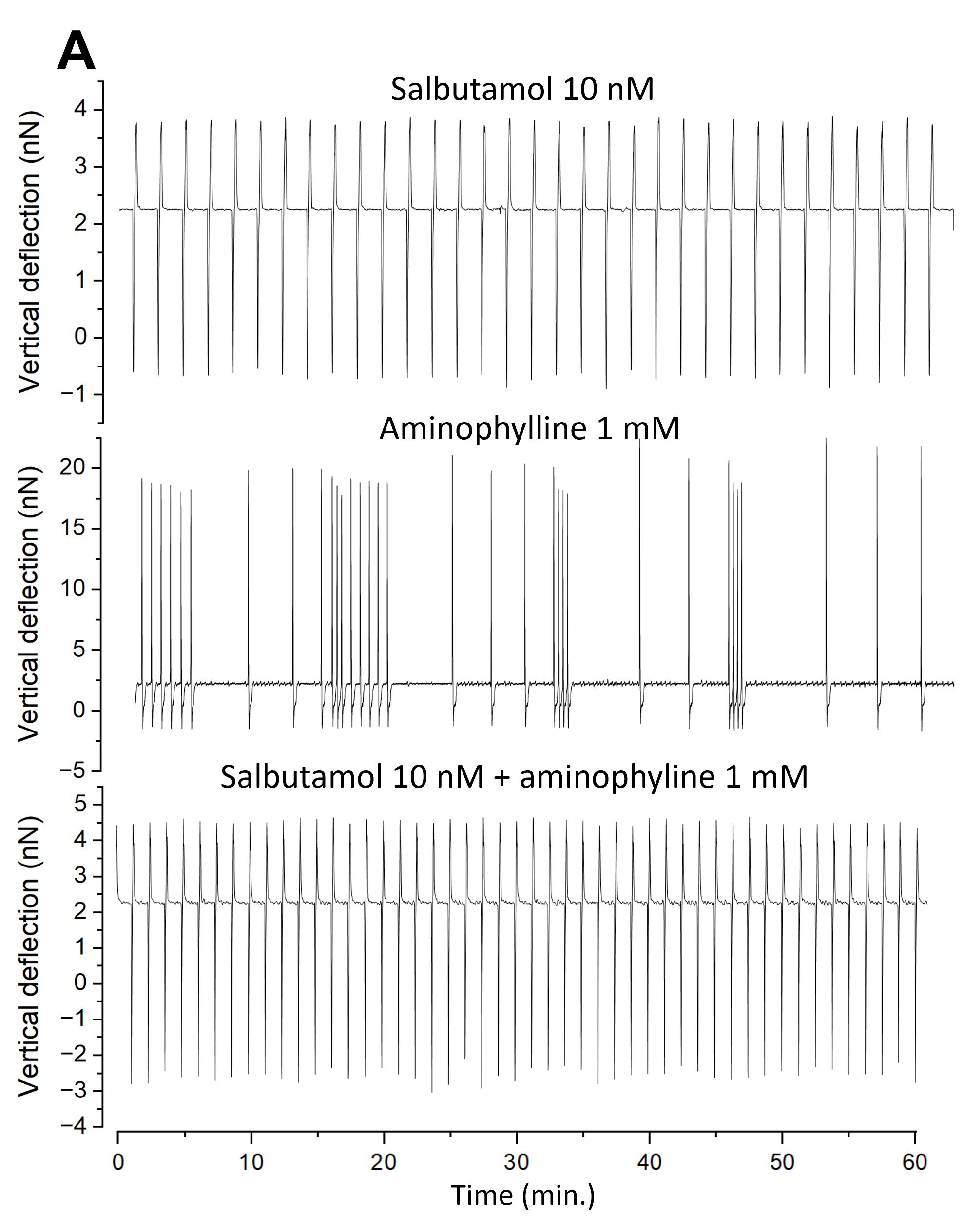

Supplement: Supplementary file 6 — Supplementary Material 6 [file 41598_2024_76846_MOESM6_ESM.tif]

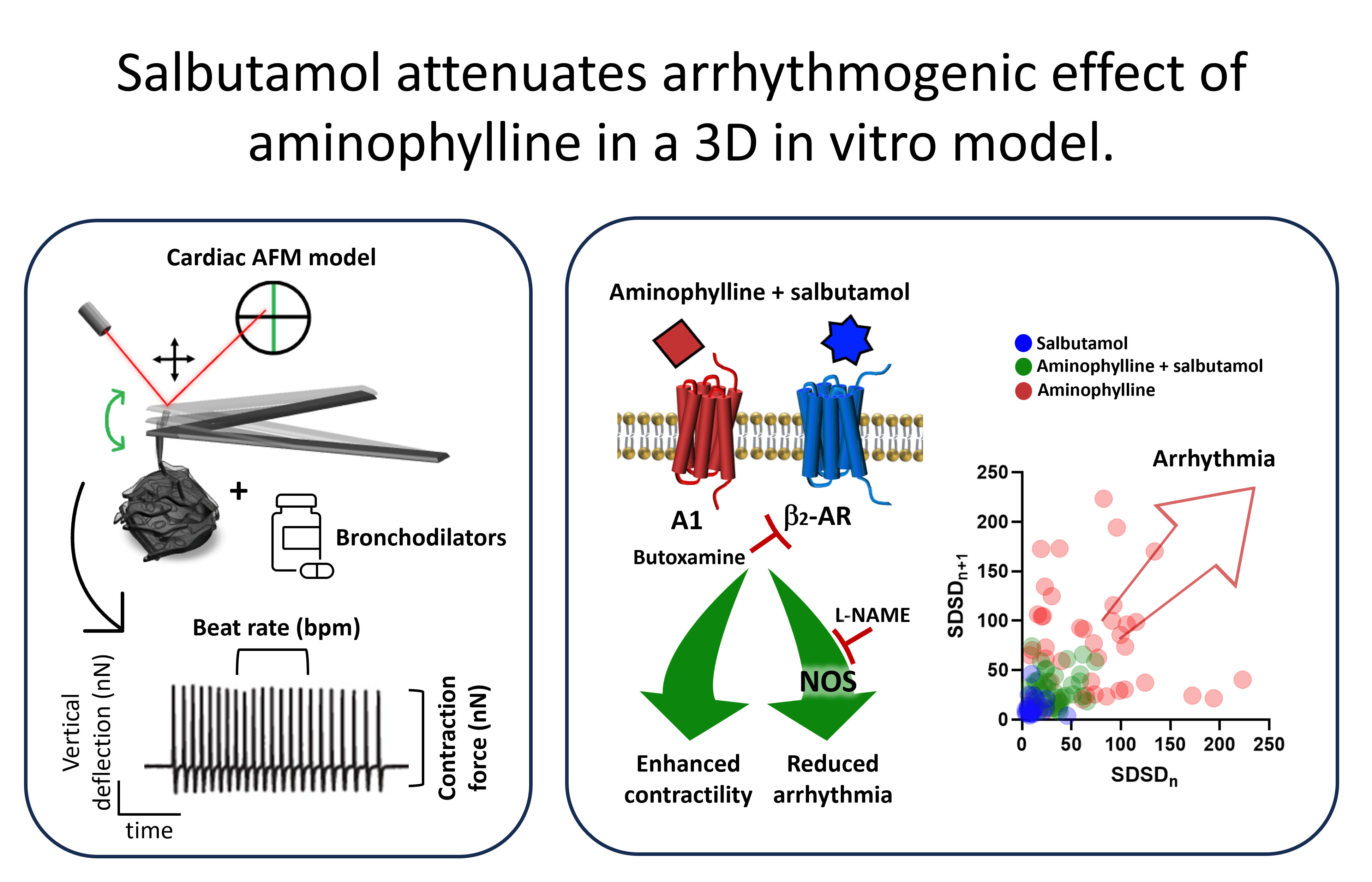

Supplement: Supplementary file 7 — Supplementary Material 7 [file 41598_2024_76846_MOESM7_ESM.tif]
